# *Pseudomonas aeruginosa* rhamnolipids stabilize human rhinovirus 14 virions

**DOI:** 10.1128/jvi.00931-25

**Published:** 2025-08-18

**Authors:** Joshua J. Baty, Aidan K. Drozdick, Julie K. Pfeiffer

**Affiliations:** 1 Department of Microbiology, University of Texas Southwestern Medical Center 12334, Dallas, Texas, USA; University of Kentucky College of Medicine, Lexington, Kentucky, USA

**Keywords:** rhinovirus, *P. aeruginosa*, virion stability, virus-microbiota interactions

## Abstract

Many mammalian viruses encounter bacteria and bacterial molecules over the course of infection. Previous work has shown that the microbial ecology of the gut plays an integral role in poliovirus and coxsackievirus infection, where bacterial glycans can facilitate virus-receptor interactions, enhance viral replication, and stabilize viral particles. However, how airway bacteria alter respiratory viral infection is less understood. Therefore, we investigated whether a panel of airway bacteria affects rhinovirus stability. We found that *Pseudomonas aeruginosa,* an opportunistic airway pathogen, protects human rhinovirus 14 (HRV14) from acid or heat inactivation. Further investigation revealed that *P. aeruginosa* rhamnolipids, glycolipids with surfactant properties, are necessary and sufficient for stabilization of rhinovirus virions. However, airway bacteria did not stabilize HRV16, a distantly related rhinovirus with higher capsid stability. Taken together, this work demonstrates that specific molecules produced by an opportunistic airway pathogen can influence a respiratory virus.

**IMPORTANCE:**

Bacteria can enhance viral stability and infection for enteric members of the *Picornaviridae*, such as poliovirus and coxsackievirus; however, whether bacteria influence respiratory picornaviruses is unknown. In this study, we examined the impacts of airway bacteria on rhinovirus, a major etiological agent of the common cold. We found that *Pseudomonas aeruginosa* protects human rhinovirus 14 from both acid and heat inactivation through rhamnolipids. Overall, this work demonstrates bacterial effects on respiratory viruses through specific bacterial molecules.

## INTRODUCTION

Rhinoviruses are the most common cause of the common cold (1–3). Rhinoviruses are a large and diverse group of enteroviruses that are divided into three species that bind various receptors—ICAM-1, LDLR, or CDHR3—that are found in the airway (2, 3). Although most rhinovirus infections are mild and self-limiting, severe and long-term consequences are possible. Rhinoviruses are the most common viral infection in those with cystic fibrosis (CF) and contribute to exacerbations (4–14). CF disease is the result of an ion imbalance at the cell surface (15), leading to aggregation of thick, sticky mucus and chronic colonization of opportunistic bacterial pathogens (16–18).

Previous work from our lab has shown that intestinal bacteria bind related enteroviruses such as poliovirus and coxsackievirus (19–23). Bacteria-virus interactions stabilize these viruses and protect them from heat inactivation (19). Furthermore, bacteria promote viral replication *in vivo*, as demonstrated by reduced titers of poliovirus and coxsackievirus in antibiotic-treated animals (22). Similarly, intestinal viruses in other families also benefit from bacteria, including mouse mammary tumor virus, murine norovirus, and certain strains of reovirus (21, 24–28). Although these interactions have been examined for these enteric viruses and related enteroviruses such as poliovirus and coxsackievirus, whether bacteria influence rhinovirus infections is unknown.

To determine if respiratory bacteria stabilize rhinovirus, we incubated human rhinovirus 14 (HRV14) and human rhinovirus 16 (HRV16) with a panel of respiratory bacteria at an inactivating acidic pH or inactivating heat and found that *Pseudomonas aeruginosa*, a notorious CF pathogen, protects HRV14, but not HRV16, from inactivation. Mechanistically, we found that rhamnolipids, biosurfactants produced by *P. aeruginosa,* are necessary and sufficient for this stabilization. Taken together, these results demonstrate that specific molecules from a ubiquitous bacterium can stabilize HRV14.

## RESULTS

### *P. aeruginosa* stabilizes HRV14

**Fig 1:**
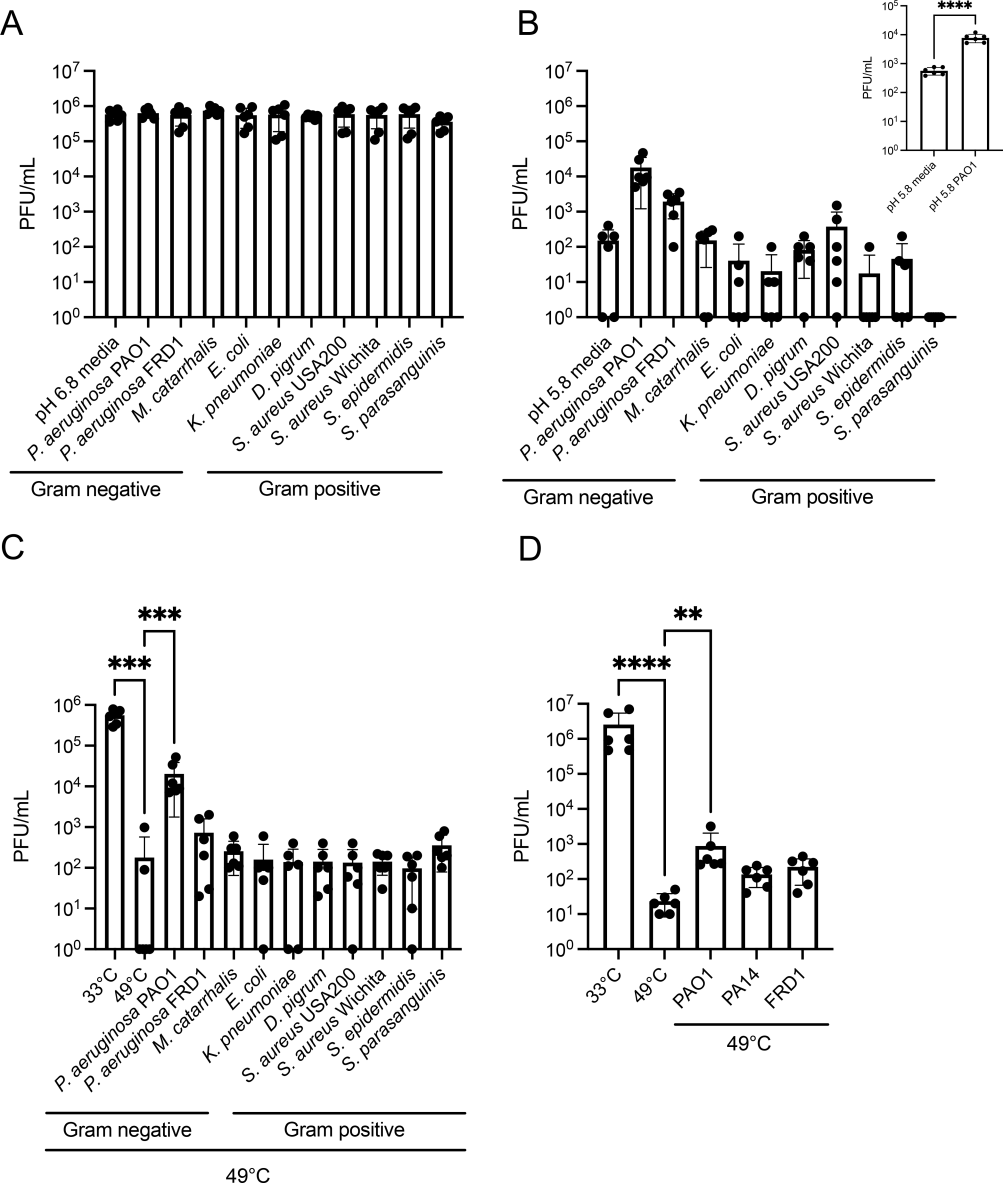
*P. aeruginosa* stabilizes HRV14. (A–C) HRV14 (10^5^ PFU) was incubated for 1 hour at a pH of 6.8 (**A**) or 5.8 (**B**) or for 2 hours at 33°C or 49°C (**C**) in the presence or absence of a panel of airway bacteria (10^6^–10^8^ CFU). Samples were centrifuged, and PFU were quantified from the supernatant by plaque assay. (**D**) HRV14 was incubated in the presence or absence of *P. aeruginosa* PAO1, PA14, or FRD1 at 33°C or 49°C for 2 hours prior to plaque assay. *n* = 6, three biological replicates with two technical replicates. **, *P* < 0.01, ***, *P* < 0.001, and ****, *P* < 0.0001 (A–D Kruskal-Wallis, Dunnett’s post hoc test, B insert unpaired *t* test).

Given that rhinoviruses likely encounter airway bacteria during infection, we questioned whether airway bacteria influence viral infection. In contrast to many other enteroviruses, rhinoviruses are acid sensitive (29). The healthy upper airway has an acidic pH that increases from the nares through the nose and sinuses with pHs of 5.5–6.5, respectively (30). The healthy lower airway has a neutral pH between 7.0 and 7.5 (31, 32). However, in the presence of inflammation, the respiratory tract pH can decrease. During asthma exacerbations, exhaled breath condensate falls to 5.2 (33). In those with CF, exhaled breath condensate is reduced to 5.8 basally and to 5.3 during exacerbations (34). To examine potential effects of bacteria on HRV14 pH sensitivity, we first incubated 10^5^ PFU HRV14 in synthetic nasal media (SNM) (35) at a pH of 6.8 or 5.8 for 1 hour before quantifying titer by plaque assay using H1 HeLa cells (Fig. 1A and B). As expected, we found a >1,000-fold reduction in viral titer at pH 5.8 (Fig. 1B) compared with pH 6.8 (Fig. 1A).

Next, we repeated the assay in the presence of a panel of airway bacteria. Many of the bacteria used in this screen (e.g., *Moraxella catarrhalis*,* Dolosigranulum pigrum*,* Staphylococcus aureus*, and* Staphylococcus epidermidis*) are commonly found in the upper airways from the nares to the sinuses (36, 37). However, some of these bacteria are enriched in the upper airways and colonize the lower airways during chronic pulmonary diseases such as CF (e.g., *P. aeruginosa*,* S. aureus*, and* Streptococcus parasanguinis*) (38, 39). Chronic pulmonary infections have high bacterial loads, with the CF lung harboring 10^7^–10^10^ CFU/mL (40). Overnight cultures of bacteria (10^6^–10^8^ CFU [Table 1]) were washed and resuspended in media at a pH of either 5.8 or 6.8, 10^5^ PFU HRV14 were added, and bacteria and virus were incubated together for 1 hour at 33°C prior to plaque assay. Bacteria had no effect on rhinovirus titers at a non-inhibitory pH of 6.8 (Fig. 1A). At a pH of 5.8, HRV14 titers were reduced across all samples, with no bacterial strain significantly protecting HRV14 from acid inactivation (Fig. 1B), although *P. aeruginosa* strains had increased yields that were not statistically significant in this initial broad screen. We repeated the pH 5.8 stability assay for HRV14 incubated with *P. aeruginosa* PAO1 and found that it significantly increased viral stability by 10-fold (Fig. 1B inset).

**TABLE 1:** Bacterial strains and plasmids

Strain	Characteristics	Overnight CFU/mL	Reference/source
*P. aeruginosa* PAO1	Wound, lab isolate	10^8^	41, 42
PAO1 *rhlA*	Transposon insertion in *rhlA*	10^8^	41
PAO1 *rhlB*	Transposon insertion in *rhlB*	10^8^	41
PAO1 *rhlC*	Transposon insertion in *rhlC*	10^8^	41
PAO1 *rhlAB^+^*	Complemented *rhlA* with *rhlAB* genes	10^8^	This study
*P. aeruginosa* FRD1	Cystic fibrosis, lab isolate	10^8^	43
*P. aeruginosa* PA14	Wild type, lab isolate	10^8^	44
*M. catarrhalis*	Wild type	10^6^	45
*Escherichia coli* K12	Lab isolate	10^8^	21
*Klebsiella pneumoniae* NCTC 9633	Lab isolate	10^8^	46
*D. pigrum*	Wild type	10^6^	47
*S. aureus* USA200	Methicillin-resistant *Staphylococcus aureus*	10^8^	48
*S. aureus* Wichita	Wound, lab isolate	10^8^	49
*S. epidermidis*	Wild type	10^8^	50
*S. parasanguinis* FW213	Wild type	10^8^	51
*E. coli* TOP10	Host strain for cloning	NA^*a*^	Thermo Fisher
pBKJB1	pBluescript K(+) ligated to *rhlAB*	NA	This study

^*a*^ NA, not applicable; tools to build complement strain.

Virion inactivation from exposure to low pH or heat occurs through premature RNA release; therefore, we used heat as an independent treatment to examine virion inactivation and potential rescue through bacteria. For these experiments, HRV14 was incubated at 33°C or 49°C for 2 hours, followed by titer analysis via plaque assay. As expected, HRV14 titers were reduced by >1,000-fold after incubation at 49°C (Fig. 1C). In the presence of airway bacterial strains, only *P. aeruginosa* PAO1 significantly increased HRV14 titers at 49°C (Fig. 1C). We next examined whether increased HRV14 viability in the presence of *P. aeruginosa* was unique to the strain PAO1 or if other strains of *P. aeruginosa* conferred protection. We compared *P. aeruginosa* strains PAO1, PA14, and FRD1. All three of these strains are typical lab strains of *P. aeruginosa*; however, exopolysaccharide and virulence factor production vary (52–54). We found that the PAO1 strain significantly increased HRV14 recovery after heat exposure, but PA14 and FRD1 strains did not (Fig. 1D), suggesting that PAO1 stabilizes HRV14 more than other strains of *P. aeruginosa.*

### HRV14 does not have increased binding to *P. aeruginosa*

**Fig 2:**
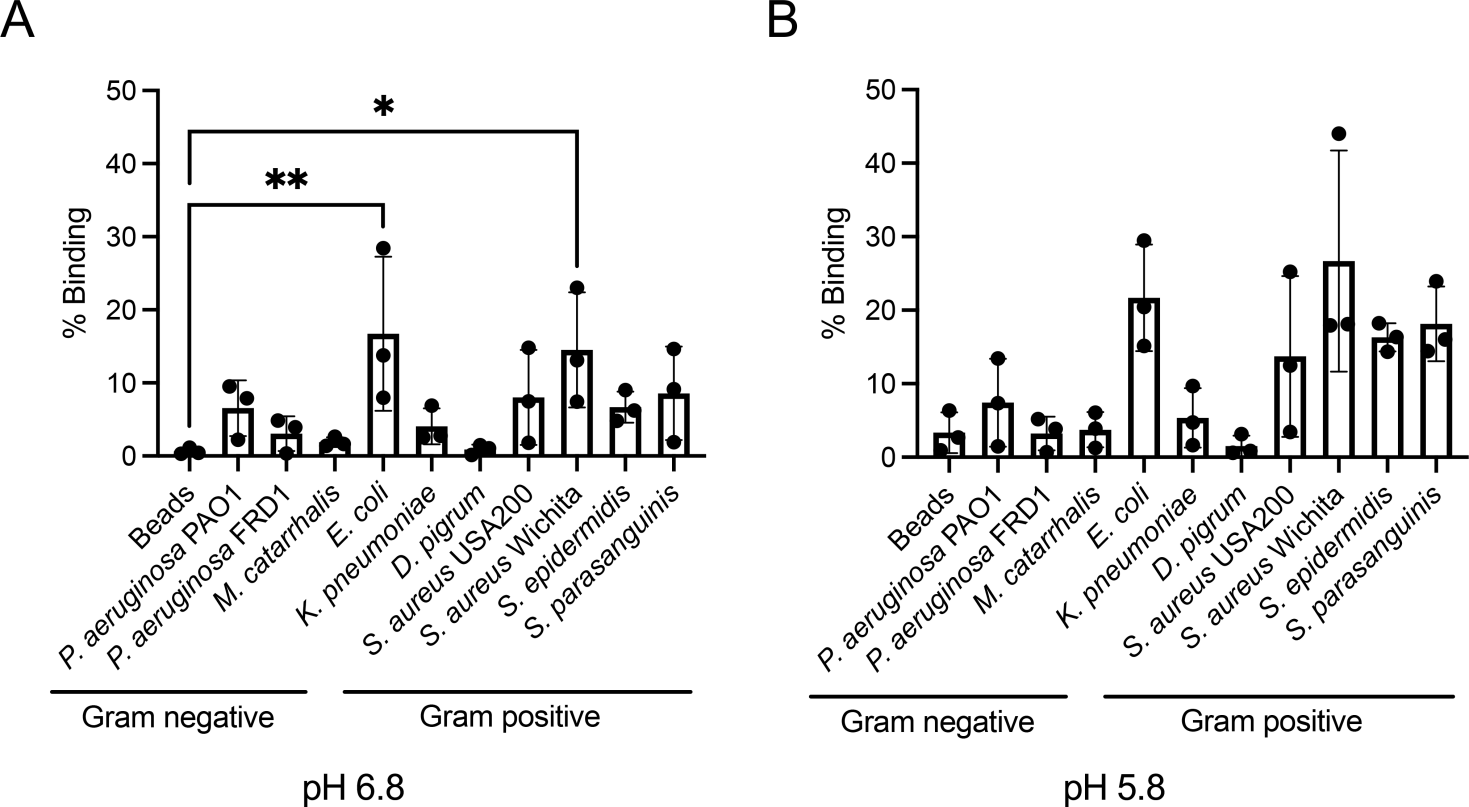
HRV14 does not have enhanced binding to *P. aeruginosa.* (A and B) ^35^S-radiolabeled HRV14 (~4,000 CPM/10^6^ PFU) was incubated in the presence or absence of streptavidin beads (2.8 µm) or 10^6^–10^8^ CFU bacteria in media at a pH of 6.8 (**A**) or 5.8 (**B**) at 33°C for 1 hour. Samples were centrifuged and washed to remove unbound virus. Bound virus was quantified via scintillation counting and normalized to input. *n* = 3. (**A**) ns, *P* > 0.05 (Kruskal-Wallis, Dunnett’s post hoc test). (**B**) *, *P* < 0.05 and **, *P* < 0.01 (one-way ANOVA, Dunnett’s post hoc test).

Our group previously reported that direct binding to bacteria and bacterial glycans stabilizes related picornaviruses such as poliovirus and coxsackievirus (19–22). Therefore, to determine whether *P. aeruginosa* PAO1 has increased binding to HRV14, potentially explaining its virion stabilization phenotype, we quantified the binding of purified, ^35^S-radiolabeled HRV14 to bacterial strains. We incubated our panel of airway bacteria (Table 1) with ^35^S-radiolabeled HRV14 (4,000 counts per minute [CPM]/10^6^ PFU) at pHs of 5.8 or 6.8 to determine if HRV14 binds relevant airway bacteria. The virus was also incubated with 2.8 µm streptavidin beads to account for nonspecific binding. *Escherichia coli* and *S. aureus* Wichita had significantly increased HRV14 binding compared to the bead control at a pH of 6.8 (Fig. 2A), although no significant differences in binding were observed at pH 5.8 (Fig. 2B). Surprisingly, HRV14 did not display enhanced binding to *P. aeruginosa*, suggesting that direct binding may not be a major facet of stabilization against acid inactivation.

### Heat-killed *P. aeruginosa* stabilizes HRV14

**Fig 3:**
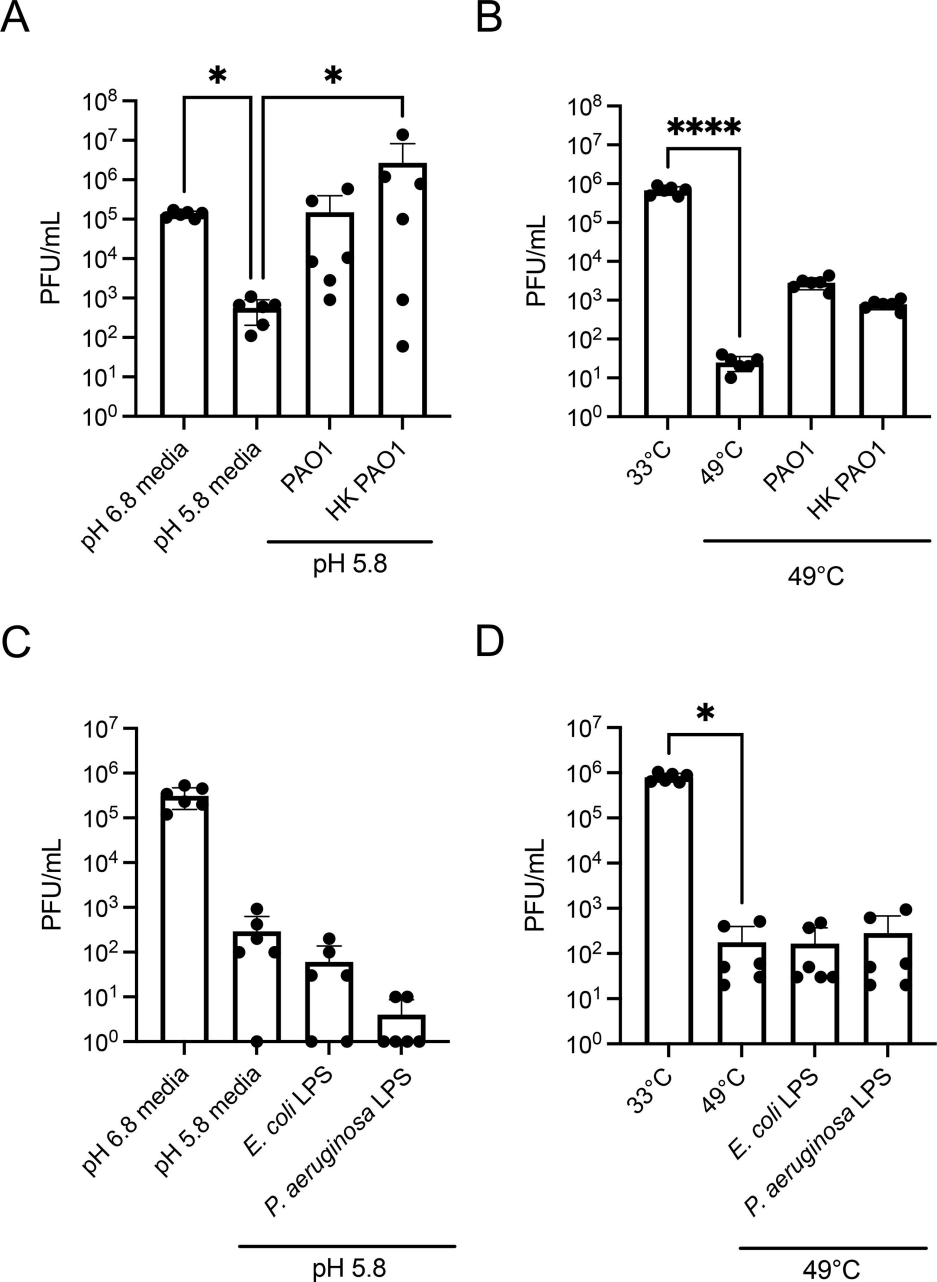
Heat-killed *P. aeruginosa* stabilizes HRV14. (A and B) HRV14 (10^5^ PFU) was incubated in the presence or absence of 10^8^ CFU live or heat-killed (HK) *P. aeruginosa* PAO1 at a pH of 5.8 or 6.8 at 33°C for 1 hour (**A**) or 33°C or 49°C for 2 hours (**B**) prior to plaque assay. (C and D) HRV14 was incubated in the presence or absence of 1 mg/mL lipopolysaccharide (LPS) from *E. coli* or *P. aeruginosa* at a pH of 5.8 or 6.8 for 1 hour (**C**) or 33°C or 49°C for 2 hours (**D**) prior to plaque assay. *n* = 6, three biological replicates with two technical replicates. *, *P* < 0.05 and ****, *P* < 0.0001 (A, C, and D, Kruskal-Wallis, Dunnett’s post hoc test, B, one-way ANOVA, Dunnett’s post hoc test).

To determine if *P. aeruginosa*-mediated protection of HRV14 from acid and heat inactivation was due to a heat-sensitive factor or relied upon active *P. aeruginosa* metabolism, HRV14 was incubated with live or heat-killed *P. aeruginosa* at a pH of 6.8 vs 5.8 (Fig. 3A) or at 33°C vs 49°C (Fig. 3B). Heat-killed *P. aeruginosa* protected HRV14 from acid inactivation, suggesting that a heat-stable *P. aeruginosa* factor stabilizes HRV14 (Fig. 3A).

Given that heat-killed *P. aeruginosa* was sufficient to protect HRV14 from acid inactivation, and our past work demonstrated that heat-stable bacterial lipopolysaccharide (LPS) stabilizes picornaviruses, we hypothesized that LPS stabilizes HRV14. As an external glycan moiety on gram-negative bacterial surfaces, LPS is a common factor that rhinovirus is likely to encounter. Previous work from our lab demonstrated that poliovirus binds LPS and that binding to LPS stabilizes poliovirus, Aichivirus, and coxsackievirus (19, 22). Conversely, LPS destabilizes enveloped influenza virions as well as alphavirus and flavivirus virions (55, 56). To assess LPS effects, HRV14 was incubated with LPS isolated from *E. coli* or *P. aeruginosa* at a pH of 5.8 vs 6.8 for 1 hour (Fig. 3C) or at 33°C vs 49°C for 2 hours (Fig. 3D), followed by plaque assay. Surprisingly, LPS did not protect HRV14 from acid or heat, suggesting that some other *P. aeruginosa* factor is responsible for stabilization.

### Rhamnolipids stabilize HRV14

**Fig 4:**
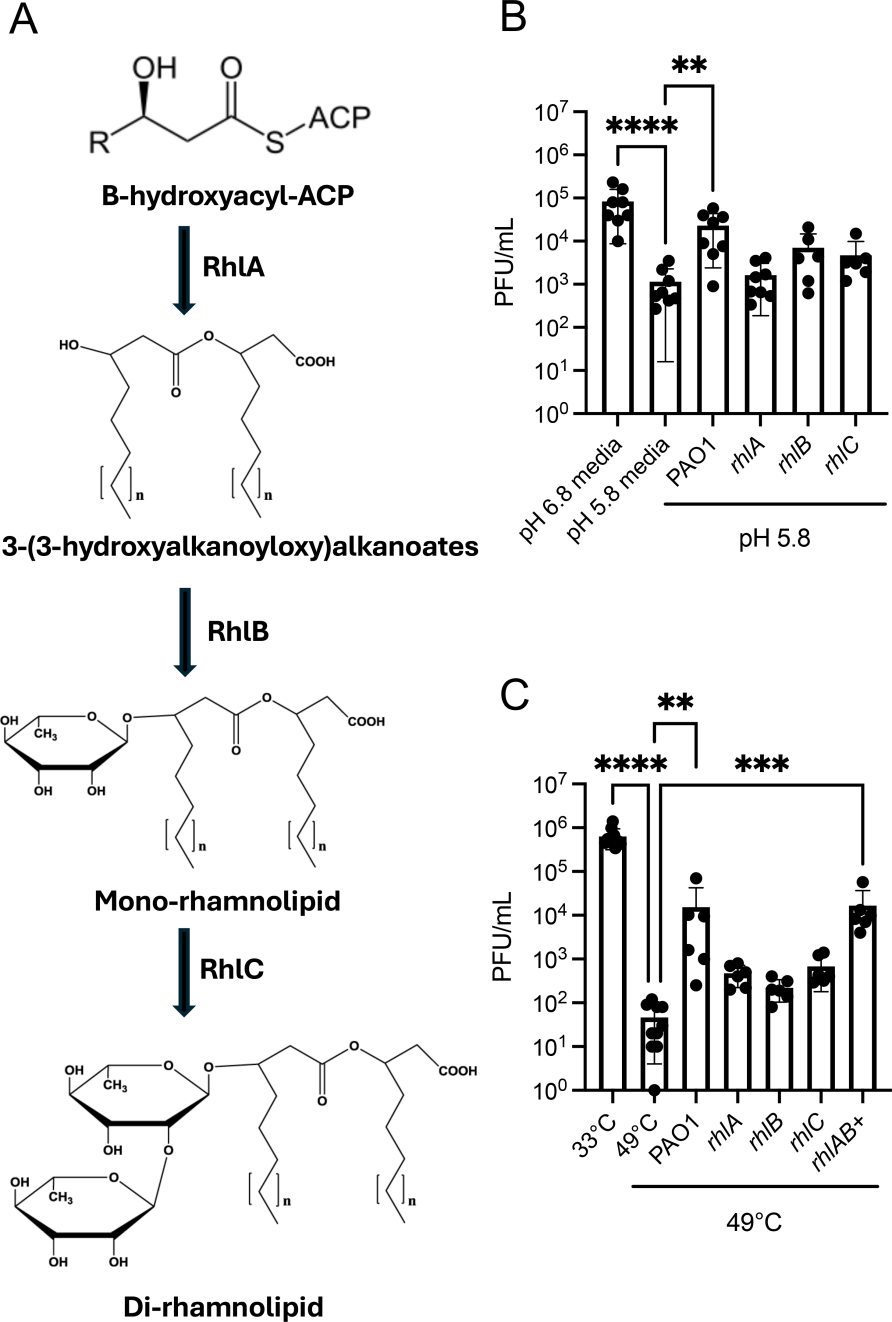
Insertion mutation of rhamnolipid synthesis genes ablates HRV14 stabilization. (**A**) *P. aeruginosa* rhamnolipid synthesis pathway. (B and C) HRV14 (10^5^ PFU) was incubated in the presence or absence of 10^8^ CFU PAO1, *rhlA*,* rhlB*,* rhlC* transposon insertion mutants, or the *rhlAB+* complement at a pH of 5.8 or 6.8 at 33°C for 1 hour (**B**) or 33°C or 49°C for 2 hours (**C**) prior to plaque assay. *n* = 6–8, 3–4 biological replicates with two technical replicates. *, *P* < 0.05, **P < 0.01, and ****, *P* < 0.0001 (Kruskal-Wallis, Dunnett’s post hoc test).

We next hypothesized that other heat-stable, high-abundance *P. aeruginosa* molecules stabilize HRV14. Like LPS, rhamnolipids are glycolipids that are produced by *P. aeruginosa* at high concentrations (57, 58). Rhamnolipids are almost exclusively produced by *P. aeruginosa*, and *P. aeruginosa* is the only bacterium in our screen that produces rhamnolipids. Rhamnolipids are important for biofilm formation and architecture, motility, and protection from phagocytosis (59–62). Rhamnolipids are synthesized by the enzymes RhlA, RhlB, and RhlC (Fig. 4A) (63). RhlA catalyzes the conversion of B-hydroxyacyl-ACP into the fatty acid dimer 3-(3-hydroxyalkanoyloxy)alkanoates (HAA) (64, 65). RhlB is a rhamnosyltransferase that catalyzes a reaction between HAA and dTDP-L-rhamnose to produce mono-rhamnolipids (66). RhlC acts as a second rhamnosyltransferase that catalyzes the conversion of mono-rhamnolipids and dTDP-L-rhamnose to di-rhamnolipids (67).

**Fig 5:**
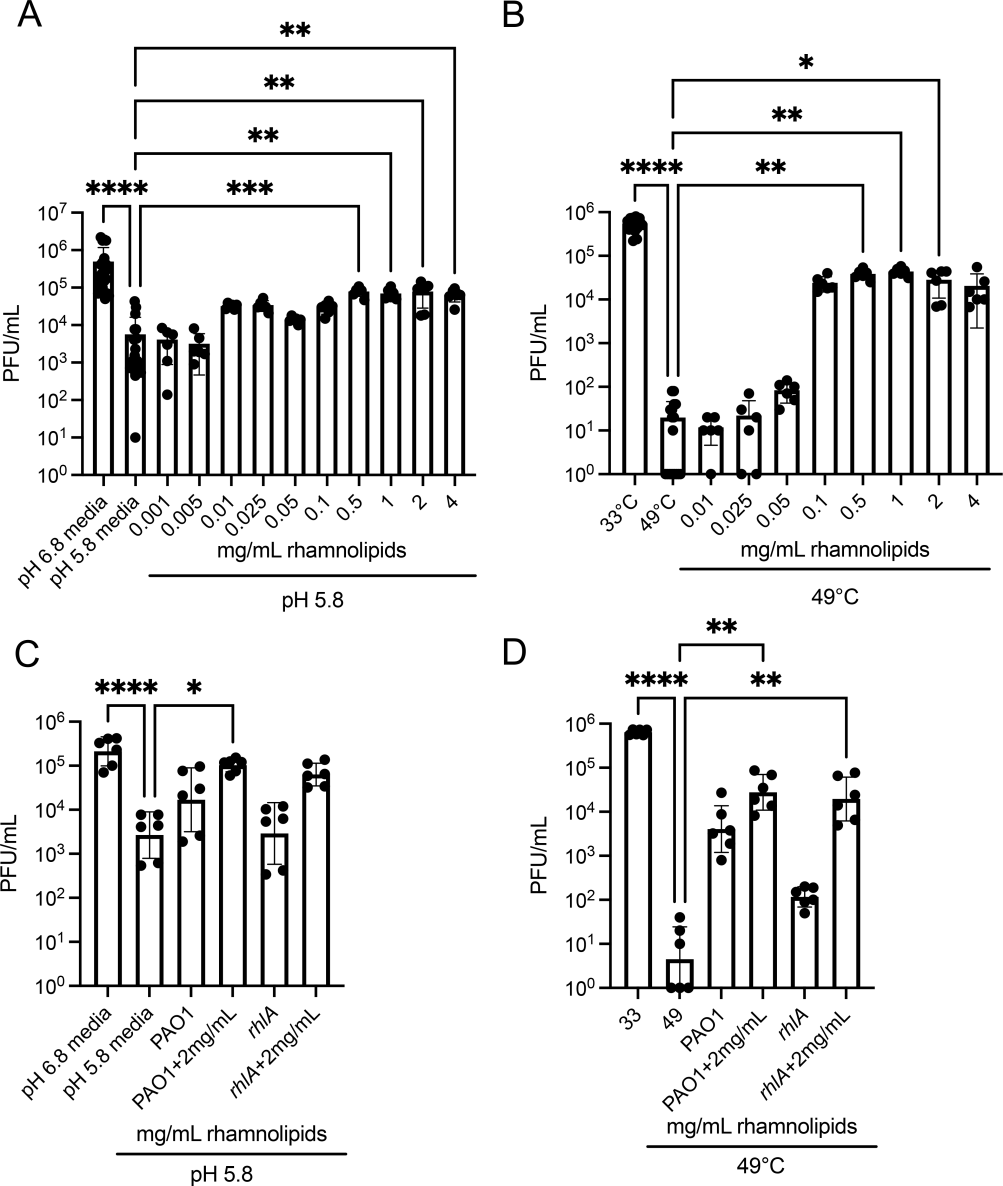
Rhamnolipids stabilize HRV14. (A and B) HRV14 (10^5^ PFU) was incubated in the presence or absence of various concentrations of rhamnolipids at a pH of 5.8 or 6.8 at 33°C for 1 hour (**A**) or 33°C or 49°C for 2 hours (**B**) prior to plaque assay. (C and D) HRV14 (10^5^ PFU) was incubated in the presence or absence of PAO1 or the *rhlA* mutant with or without exogenous rhamnolipids at a pH of 5.8 or 6.8 at 33°C for 1 hour (**C**) or 49°C for 2 hours (**D**) prior to plaque assay. *n* = 6, three biological replicates with two technical replicates. *, *P* < 0.05, **, *P* < 0.01, ***, *P* < 0.001, and ****, *P* < 0.0001 (A, B, and D Kruskal-Wallis, Dunnett’s post hoc test, C, one-way ANOVA, Dunnett’s post hoc test).

To examine the potential impact of *P. aeruginosa* rhamnolipids on HRV14 stabilization, we used strains with transposon insertions within rhamnolipid synthesis genes to test for necessity (*rhlA*,* rhlB*, and* rhlC*), and we used exogenous rhamnolipids to test for sufficiency. The *rhlA*,* rhlB*, and *rhlC* mutants in the *P. aeruginosa* PAO1 background (41) were incubated with HRV14 at a pH of 5.8 vs 6.8 for 1 hour (Fig. 4B) or at 33°C vs 49°C for 2 hours (Fig. 4C), followed by plaque assay. All mutants in the rhamnolipid synthesis pathway failed to protect HRV14 from acid and heat inactivation (Fig. 4B and C). To examine whether loss of virion stabilization by the *rhlA* strain was a direct result of the transposon insertion rather than off-target effects, we determined whether heat stabilization of HRV14 was restored in an *rhlA* strain complemented with *rhlAB* expression in *trans* (*rhlAB^+^*). In the presence of heat, the *rhlAB^+^* complement restored protection, suggesting that rhamnolipids are necessary for this phenotype. Next, we tested whether purified rhamnolipids could stabilize HRV14 in the absence of bacteria. HRV14 was incubated with various concentrations of rhamnolipids at a pH of 5.8 vs 6.8 for 1 hour (Fig. 5A) or at 33°C vs 49°C for 2 hours (Fig. 5B), followed by plaque assay. Rhamnolipids protected HRV14 from both acid and heat inactivation at a concentration of 0.5 mg/mL. We next examined whether the addition of exogenous rhamnolipids could rescue HRV14 virions from acid or heat when incubated with the *rhlA* mutant. While the *rhlA* mutant did not increase viral titer in the presence of acid (Fig. 5C) or heat (Fig. 5D), the addition of rhamnolipids increased HRV14 PFU, further suggesting that rhamnolipids are sufficient for HRV14 stabilization. Overall, data in Fig. 4 and 5 indicate that rhamnolipids are necessary and sufficient for stabilization of HRV14 by *P. aeruginosa*.

**Fig 6:**
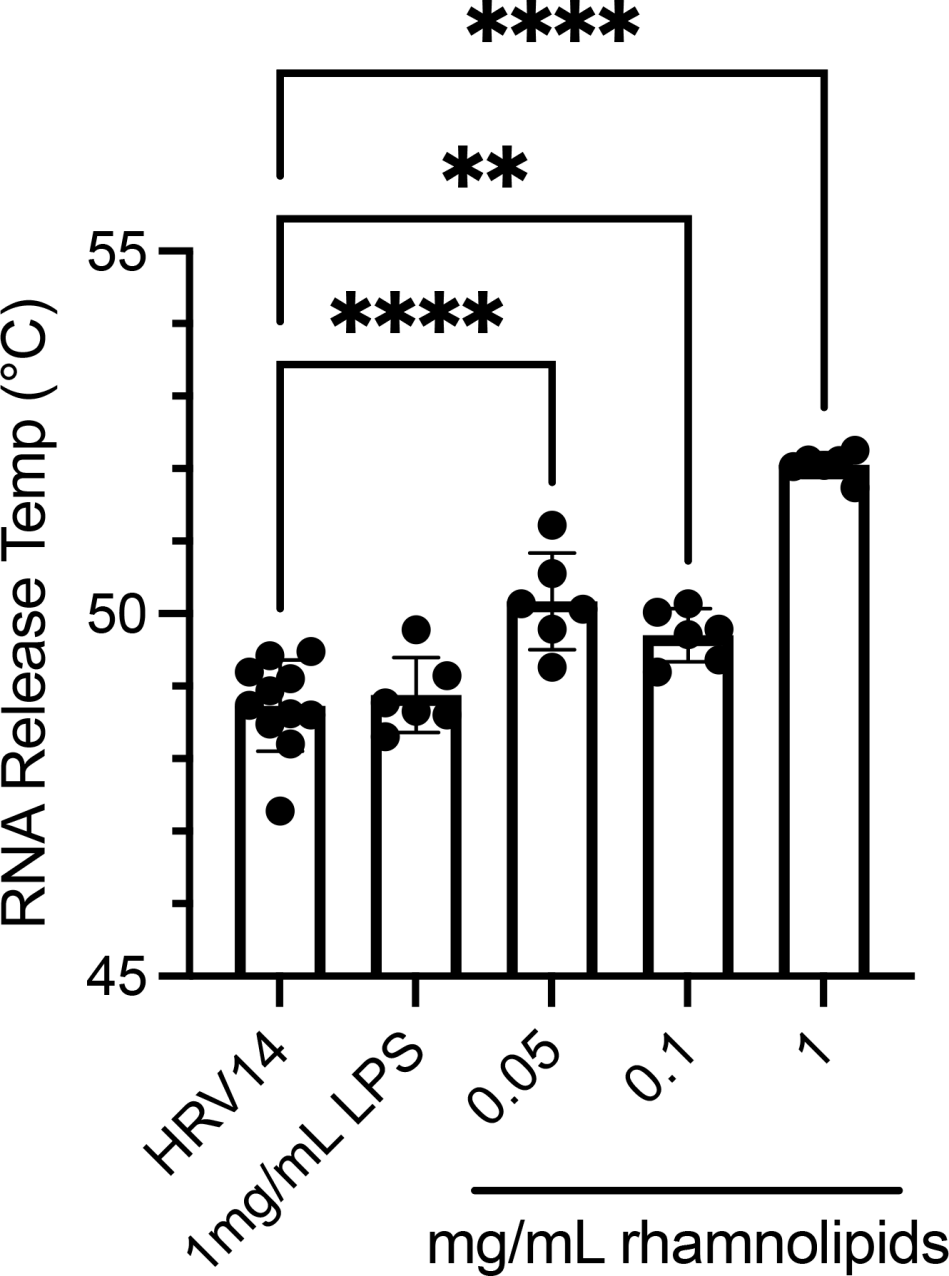
Rhamnolipids enhance HRV14 thermostability. HRV14 thermostability profile using a cell-free PaSTRy. HRV14 (10^5^ PFU) was added to SYBR green II with or without LPS or rhamnolipids. Samples were heated from 25°C to 95°C on a 1% stepwise gradient with fluorescence monitoring. *n* = 6, three biological replicates with two technical replicates. **, *P* < 0.01 and ****, *P* < 0.0001 (one-way ANOVA, Dunnett’s post hoc test).

To confirm that rhamnolipids stabilize HRV14 using an assay independent from viral viability assays, we performed a cell-free Particle Stability Thermal Release assay (PaSTRy) (68). Through this assay, virion RNA release is measured over a temperature gradient using SYBR green II dye to define the exact temperature of virion inactivation. RNA release was measured for HRV14 in the presence or absence of *P. aeruginosa* LPS (as a negative control) or rhamnolipids (Fig. 6). Untreated HRV14 released RNA at 48.7°C. As expected from our plaque-based assays, LPS had no effect on the temperature at which HRV14 RNA release occurred. However, rhamnolipids shifted HRV14 RNA release temperatures by ~1°C at 0.05 and 0.1 mg/mL concentrations and by ~3°C at a 1 mg/mL concentration. Taken together, these results demonstrate that rhamnolipids stabilize HRV14.

### Respiratory bacteria do not stabilize HRV16

**Fig 7:**
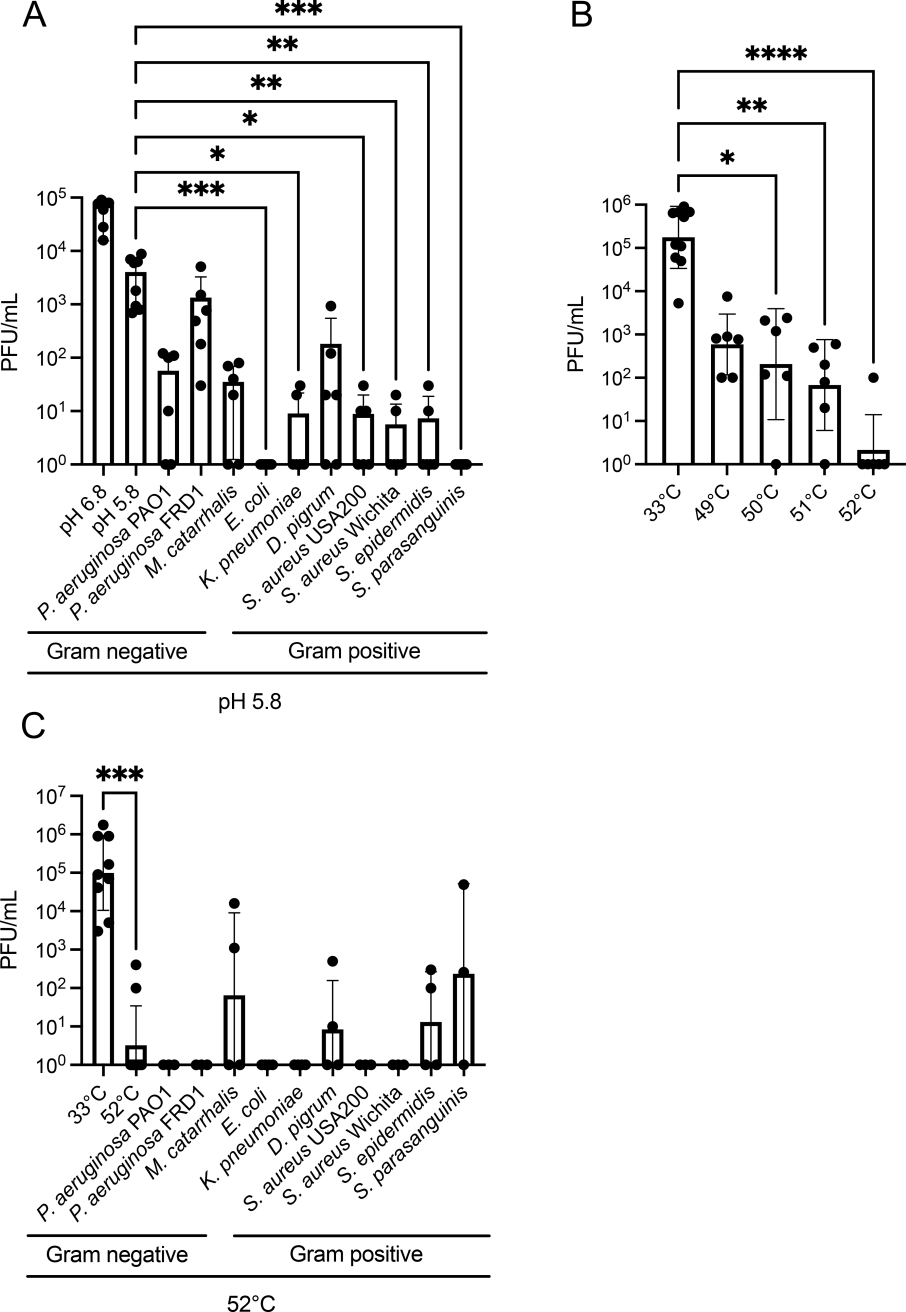
Respiratory bacteria do not stabilize HRV16. (**A**) HRV16 (10^5^ PFU) was incubated for 1 hour at a pH of 6.8 or 5.8 in the presence or absence of a panel of airway bacteria (10^6^–10^8^ CFU) prior to plaque assay. (**B**) HRV16 (10^5^ PFU) was incubated at 33°C, 49°C, 50°C, 51°C, or 52°C for 2 hours prior to plaque assay. (**C**) HRV16 (10^5^ PFU) was incubated at 33°C or 49°C in the presence or absence of a panel of airway bacteria for 2 hours prior to plaque assay. *n* = 6, three biological replicates with two technical replicates. *, *P* < 0.05, **, *P* < 0.01, ***, *P* < 0.001, and ****, *P* < 0.0001 (Kruskal-Wallis, Dunnett’s post hoc test).

The rhinovirus genus encompasses roughly 100 distinct strains from three different species (3). Given the high degree of divergence between rhinovirus strains, we were curious whether other rhinoviruses are also stabilized by respiratory bacteria. Thus, we chose to test HRV16, a virus distantly related to HRV14. HRV16 is a species A rhinovirus, while HRV14 is a B species rhinovirus. Both HRV14 and HRV16 bind the same receptor, ICAM-1, but their capsid amino acid sequences differ by 49% (69–71). Thus, we incubated HRV16 at a pH of 5.8 or 6.8 at 33°C for 1 hour in the presence of airway bacteria and performed a plaque assay. Notably, HRV16 PFU was decreased by 20-fold at a pH of 5.8 compared to 6.8 (Fig. 7A), whereas HRV14 PFU was decreased by 4,000-fold (Fig. 1A and B), suggesting that HRV16 is more stable than HRV14. In contrast to HRV14, no bacterium protected HRV16 from acid inactivation, with many bacteria significantly enhancing inactivation.

As HRV16 was more stable than HRV14 in the presence of acid, we determined whether HRV16 also has increased stability in the presence of heat. We incubated HRV16 at 33°C, 49°C, 50°C, 51°C, or 52°C for 2 hours and performed a plaque assay. At 49°C, HRV16 titer was decreased by 200-fold, while HRV14 titer was decreased by 3,000-fold (Fig. 7B and 1C). A comparable reduction in HRV16 titer was only observed at 52°C, suggesting that HRV16 is more heat stable than HRV14. We next incubated HRV16 at either 33°C or 52°C in the presence of airway bacteria. As we observed with the acid incubation, no bacterium protected HRV16 from heat inactivation (Fig. 7C).

## DISCUSSION

Rhinoviruses are important respiratory pathogens, but the potential impacts of bacteria on rhinovirus infection are largely unknown. Here, we screened a panel of respiratory bacteria and found that *P. aeruginosa*, an opportunistic pathogen that establishes chronic infections in those with chronic airway diseases, protects HRV14, but not HRV16, from acid and heat inactivation. Investigation of *P. aeruginosa* strains deficient for rhamnolipid production and addition of exogenous rhamnolipids revealed that rhamnolipids were necessary and sufficient for HRV14 stabilization.

Rhamnolipids are glycolipids that are important for *P. aeruginosa* physiology and infection. Rhamnolipids are almost exclusively produced by *P. aeruginosa*, and *P. aeruginosa* is the only bacterium in our panel that produces rhamnolipids. *P. aeruginosa* can produce copious amounts of rhamnolipids, with wild-type *P. aeruginosa* PAO1 producing as much as 39 mg/mL (57)—which is nearly 80-fold more than what was required for HRV14 stabilization (Fig. 5A and B). Rhamnolipid regulation and production are complex and vary between strains. Rhamnolipid production is regulated through both the Las and Rhl quorum sensing systems (72). As *P. aeruginosa* colonizes and adapts to the CF lung environment, these systems acquire mutations that significantly alter their regulation. These changes in regulation may explain why the lab strain PAO1 significantly protected HRV14 from heat inactivation while the CF isolate FRD1 did not (Fig. 1D). Nonetheless, rhamnolipids are present in sputum samples from people with CF that are colonized with *P. aeruginosa* (73, 74). Rhamnolipids help shape biofilm architecture (60), mediate *P. aeruginosa* dispersal (75–77), enhance *P. aeruginosa* motility (76), decrease phagocytosis (61, 62), and damage cell membranes (62, 78). Rhamnolipids inhibit the colonization and disperse a wide array of other bacteria (77, 79–84). Additionally, rhamnolipids inactivate enveloped viruses such as herpesviruses, coronaviruses, and respiratory syncytial virus via envelope disruption (85–89).

Less is known about interactions between rhamnolipids and nonenveloped viruses, such as rhinoviruses and other picornaviruses. Rhamnolipids have no effect on poliovirus stability (85), but *in silico* modeling of HRV14 suggests that rhamnolipids interact with the hydrophobic pocket under the canyon floor of the capsid, where rhinoviruses bind their receptors (69). This interaction may be responsible for the stabilization phenotype herein. Similar stabilization phenotypes have been observed with antivirals, such as WIN compounds, that bind the hydrophobic pocket under the canyon floor (90). This binding prevents the uncoating of rhinoviruses but also enhances thermostability and resistance to acid stress (91). Notably, while the addition of rhamnolipids enhances thermostability, HRV14 replication is not impacted, suggesting that rhamnolipids confer stability to HRV14 without inhibiting RNA uncoating during infection.

While we observed protective effects of rhamnolipids on HRV14, *P. aeruginosa* did not protect HRV16 from acid or heat, suggesting that rhamnolipids do not confer stability to HRV16 (Fig. 7). HRV16 is more stable than HRV14 (Fig. 1 and 7) (69, 92). Additionally, HRV14 demonstrates increased capsid “breathing” when compared to HRV16, where VP4, the most internal capsid protein, is spontaneously extruded and exposed to the surface (92, 93). The enhanced HRV14 capsid dynamics make HRV14 intrinsically more unstable than HRV16. Thus, HRV14 may partially overcome this stability deficit through *P. aeruginosa* rhamnolipids. However, further studies are required to fully delineate the role of rhamnolipids during rhinovirus infection.

Beyond viral stability, exposure to biosurfactants, such as rhamnolipids, can increase pathogenesis of other picornaviruses, such as encephalomyocarditis virus (EMCV) (94–96). The pesticides dichloro-diphenyl-trichloroethane and fenitrothion are associated with clusters of Reye’s Syndrome, a rare condition involving liver pathology and brain swelling that often accompanies viral infection. These surfactants increase EMCV uncoating in treated cells (95). Additionally, these compounds reduce interferon responses, contributing to increased morbidity and mortality in mice (95).

Taken together, we found that rhamnolipids, glycolipids produced by the opportunistic pathogen *P. aeruginosa*, increase the stability of HRV14. This interaction may be clinically relevant as many people with CF are chronically colonized by *P. aeruginosa*, and rhinoviruses are a common cause of exacerbation events. Future studies are necessary to determine the role rhamnolipids and other compounds play over the course of rhinovirus infection.

## MATERIALS AND METHODS

### Cells and viruses

HeLa H1 cells were propagated in Dulbecco's modified Eagle medium supplemented with 10% calf bovine serum and 1% antibiotics. Cells were grown at 37°C with 5% CO_2_. HRV14 and HRV16 were propagated from an infectious clone (gift of William Jackson), and infections were performed at 33°C 5% CO_2_.

### Bacterial strains, culture conditions, and reagents

*P. aeruginosa* PAO1, FRD1, PA14, and the PAO1 isogenic mutants* rhlA*,* rhlB*,* rhlC*, *S. aureus*,* S. epidermidis*,* E. coli*, and *Klebsiella pneumoniae* were maintained on lysogeny broth (LB) agar and grown on LB at 37°C with shaking at 250 rpm. *M. catarrhalis* was grown in brain heart infusion media at 37°C with shaking at 250 rpm. *D. pigrum* and *S. parasanguinis* were grown in Todd Hewitt broth/agar at 37°C with 5% CO_2_. The *rhlAB* complementing plasmid was generated by amplifying *rhlAB* from the PAO1 chromosome with primers: 5′-AAAAGCTTGGTACCAGCGTTTCGACACCGG-3′ and 5′-AAGAATTCCATTGGCCCGGGGTATGA-3′. The PCR product was digested with HindIII and EcoRI and cloned into pBluescript K(+) (Addgene). The *rhlAB*-containing plasmid was then transformed into PAO1 *rhlA*. PAO1 *rhlAB^+^* was selected on LB with 100 µg/mL carbenicillin and was maintained and cultured in 100 µg/mL carbenicillin. Synthetic nasal media were prepared as described in reference 35.

### Quantifying effects of bacteria on viral stability

For acid sensitivity assays, overnight cultures of bacteria (Table 1, 10^6^ or 10^8^ CFU, depending on the strain) were centrifuged, washed in SNM at a pH of either 5.8 or 6.8, centrifuged, and resuspended in SNM at a pH of either 5.8 or 6.8. HRV14 (10^5^ PFU) was added, and the virus and bacteria of interest were incubated at 33°C with 5% CO_2_ for 1 hour. No bacteria tested significantly altered the pH of the media over the course of the incubation. For heat sensitivity assays, overnight cultures were centrifuged, washed with phosphate buffered saline (PBS), and resuspended in PBS. HRV14 was added as above, and the mixture was incubated at either 33°C or 49°C for 2 hours. After each incubation, samples were centrifuged, and PFUs in the supernatants were quantified via plaque assay as described (19). Briefly, samples were diluted in PBS supplemented with 100 µg/mL CaCl_2_ and 100 µg/mL MgCl_2_ and allowed to attach to cells for 30 minutes at 33°C with 5% CO_2_. Agar overlays containing DMEM with 10% calf bovine serum and 1% antibiotics were added and removed 48 hours after infection. PFUs were enumerated following crystal violet staining of monolayers.

### Quantifying viral binding to bacterial cells

^35^S-radiolabeled HRV14 was generated as previously described (20). Briefly, infected cells were pulsed with ^35^S-amino acids to label progeny virions, cell-associated virions were collected, and purified using Capto Core 700 beads (Cytivia) according to the manufacturer’s instructions. Briefly, rhinovirus was mixed end-over-end at 4°C with capto core beads for 45-minute increments three times. The slurry was centrifuged, and the virus from the supernatant was assessed for purity by sodium dodecyl sulfate polyacrylamide gel electrophoresis (SDS-PAGE) For binding assays, ~4,000 CPM (10^6^ PFU) HRV14 was added to overnight bacterial cultures or streptavidin beads (Invitrogen, Dynabeads) resuspended in SNM pH 5.8 or 6.8. Incubation proceeded for 1 hour at 33°C with 5% CO_2_, and the mixture was centrifuged and washed to remove unbound virus. The pellet was resuspended in Budget-Solve complete counting cocktail (Research Products International), and CPM was determined by scintillation counting.

### Quantifying effects of lipopolysaccharide and rhamnolipids on viral stability

Live or heat-killed *P. aeruginosa* PAO1 was incubated with HRV14 as above. PAO1 was heat-killed by incubating at 95°C for 10 minutes. LPS (at 1 mg/mL) from *E. coli* (O111:B4, Sigma) or *P. aeruginosa* (PA-10, Sigma) was resuspended in SNM pH 5.8 or PBS and incubated at 33°C or 49°C and quantified via plaque assay as above. Exogenous rhamnolipids (a mix of mono- and di- rhamnolipids) from *P. aeruginosa* (Sigma) were added and incubated via the same scheme.

### Particle stability thermal release assay

Capto-core (Cytiva) purified and Amicon filter-concentrated (Sigma) HRV14 (~10^5^ PFU) was combined with rhamnolipids, SYBR green II (10× final concentration, Invitrogen), and buffer (10 mM HEPES at pH 8, 200 mM NaCl). The 50 µL reactions were heated from 25°C to 95°C on a 1% gradient in an ABI 7500 real-time thermocycler (Applied Biosystems) with fluorescent monitoring.

### Data analysis

All statistical analyses were performed using GraphPad Prism version 10.4.2 for macOS. Normality was assessed via the Shapiro-Wilk test. Further analyses were performed where indicated.

## Data Availability

All data are contained within the article.
